# Experimental infections and co-infections with *Leishmania braziliensis* and *Leishmania infantum* in two sand fly species, *Lutzomyia migonei* and *Lutzomyia longipalpis*

**DOI:** 10.1038/s41598-020-60600-7

**Published:** 2020-02-27

**Authors:** Joanna Alexandre, Jovana Sadlova, Tereza Lestinova, Barbora Vojtkova, Magda Jancarova, Lucie Podesvova, Vyacheslav Yurchenko, Filipe Dantas-Torres, Sinval P. Brandão-Filho, Petr Volf

**Affiliations:** 1 Department of Immunology, Aggeu Magalhães Institute, Fiocruz, Pernambuco Brazil; 2 Department of Parasitology, Faculty of Science, Charles University, Prague, Czech Republic; 3 Life Science Research Centre, Faculty of Science, University of Ostrava, Ostrava, Czech Republic; 4 Martsinovsky Institute of Medical Parasitology, Tropical and Vector Borne Diseases, Sechenov University, Moscow, Russia

**Keywords:** Parasite development, Entomology

## Abstract

Leishmaniases are neglected tropical diseases and *Leishmania* (*Leishmania*) *infantum* and *Leishmania* (*Viannia*) *braziliensis* are the most important causative agents of leishmaniases in the New World. These two parasite species may co-circulate in a given endemic area but their interactions in the vector have not been studied yet. We conducted experimental infections using both single infections and co-infections to compare the development of *L*. (*L*.) *infantum* (OGVL/mCherry) and *L*. (*V*.) *braziliensis* (XB29/GFP) in *Lutzomyia longipalpis* and *Lutzomyia migonei*. Parasite labelling by different fluorescein proteins enabled studying interspecific competition and localization of different parasite species during co-infections. Both *Leishmania* species completed their life cycle, producing infective forms in both sand fly species studied. The same happens in the co infections, demonstrating that the two parasites conclude their development and do not compete with each other. However, infections produced by *L*. (*L*.) *infantum* reached higher rates and grew more vigorously, as compared to *L*. (*V*.) *braziliensis*. In late-stage infections, *L*. (*L*.) *infantum* was present in all midgut regions, showing typical suprapylarian type of development, whereas *L*. (*V*.) *braziliensis* was concentrated in the hindgut and the abdominal midgut (peripylarian development). We concluded that both *Lu. migonei* and *Lu. longipalpis* are equally susceptible vectors for *L*. (*L*.) *infantum*, in laboratory colonies. In relation to *L*. (*V*.) *braziliensis*, *Lu. migonei* appears to be more susceptible to this parasite than *Lu. longipalpis*.

## Introduction

Leishmaniases are important parasitic diseases, causing serious medical problems in many countries, as they rank in the top-three list of neglected tropical diseases caused by protists^1^. The causative agents, flagellates of the genus *Leishmania* (Kinetoplastida: Trypanosomatidae), subgenera *Leishmania* and *Viannia*, are transmitted by phlebotomine sand flies (Diptera: Psychodidae)^2^.

In Brazil, *Leishmania* (*Leishmania*) *infantum* and *Leishmania* (*Viannia*) *braziliensis* are the most important causative agents of leishmaniases in humans^3^. *L*. (*V*.) *braziliensis* causes a typical cutaneous leishmaniasis (CL), which may progress to mucosal disease, whereas *L*. (*L*.) *infantum* infections is responsible for a life-threatening form of the disease – visceral leishmaniasis (VL). These two parasite species also differ in their development in the sand fly vector; both colonize the sand fly midgut, but only *L*. (*V*.) *braziliensis* was documented to colonize the hindgut (peripylarian development)^4^.

As different parasite species may co-circulate in a given endemic area^5^, the significance of co-infections by different *Leishmania* in sand flies is poorly understood. So far, rather limited number of investigations has been conducted to study the simultaneous development of different *Leishmania* species in the same sand fly^6,7^. In particular, it would be interesting and important to study simultaneous development of a suprapylarian *Leishmania* and a peripylarian *Viannia*.

The present study was designed to fill this gap in knowledge and conducted experimental co-infections to compare the development of *L*. (*L*.) *infantum* and *L*. (*V*.) *braziliensis* in *Lutzomyia longipalpis*, a known permissive vector^8,9^, and *Lutzomyia migonei*, which is susceptible to the development of *L*. (*V*.) *braziliensis*^10^ and to different strains of *L*. (*L*.) *infantum*^11^. Parasite labelling by different fluorescein proteins enabled us to study interspecific competition during co-infections.

## Results

### Experimental infections of *Lu. migonei*

**Figure 1:**
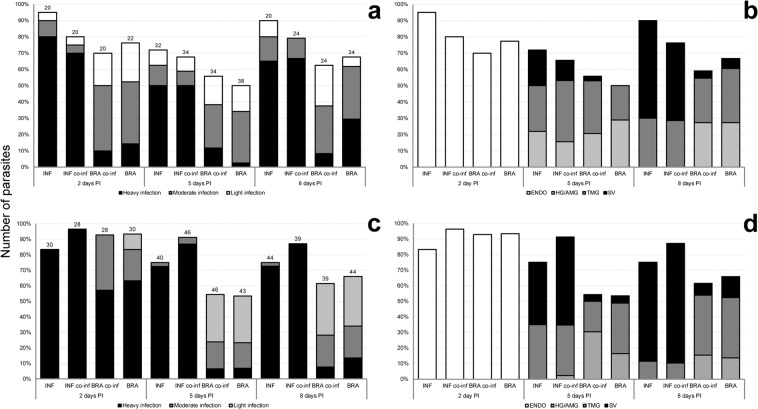
Rates and intensities (**a**,**c**) and localization of *Leishmania* spp. (**b**,**d**) in *Lu. migonei* (**a,b**) and *Lu. longipalpis* (**c,d**) evaluated by fluorescence microscopy. Intensities of infections were classified into three categories: light (<100 parasites/gut), moderate (100–1000 parasites/gut), or heavy (>1000 parasites/gut). PI = post infections, INF = *L. infantum*, BRA = *L. braziliensis*. Columns represent intensity of the *Leishmania* species either in single infections (INF single, BRA single) of in coinfection (INF co-inf, BRA co-inf). Numbers of dissected sand fly females are shown above the bars.

In total, 322 *Lu. migonei* females were dissected. On day 2 post infection (PI), *L*. (*L*.) *infantum* infection rate was 95% and parasites grew very vigorously, whereas *L*. (*V*.) *braziliensis* infection rate was 70% and parasites grew slower (Fig. 1a). Both parasite species were restricted to the endoperitrophic space (bloodmeal surrounded by peritrophic matrix) (Fig. 1b). There were no statistically significant differences in the infection rate (*X*^2^ = 4.29, *df* = 3, *P* = 0.23).

On day 5 PI, *L*. (*L*.) *infantum* infection rate was still high (Fig. 1a), parasites colonized both parts of the midgut (abdominal and thoracic midgut) and, in more than 20% of female sand flies, also the stomodeal valve (Fig. 1b). On the other hand, *L*. (*V*.) *braziliensis* infection rate was lower (50%) and parasites colonized only the midgut and the hindgut. Differences in infection rates were non-significant (*X*^2^ = 4.52, *df* = 3, *P* = 0.21), whereas differences in the location of the parasites were significant (*X*^2^ = 6.94, *df* = 1, *P* < 0.01). Similar differences between *L*. (*L*.) *infantum* and *L*. (*V*.) *braziliensis* were also observed in parasite loads and their localization during coinfections (Fig. 1b).

On day 8 PI, in late stage infections, *L*. (*L*.) *infantum* developed very successfully (infection rate 90%), whereas *L*. (*V*.) *braziliensis* grew less successfully (infection rate 68%), but there was no statistical difference (*X*^2^ = 3.44, *df* = 1, *P* = 0.06). *L* (*L*.) *infantum* colonized the cardia and the stomodeal valve significantly more frequently (*X*^2^ = 14.43, *df* = 1, *P* < 0.01), whereas *L*. (*V*.) *braziliensis* did not colonize the stomodeal valve at all (Fig. 1b).

**Figure 2:**
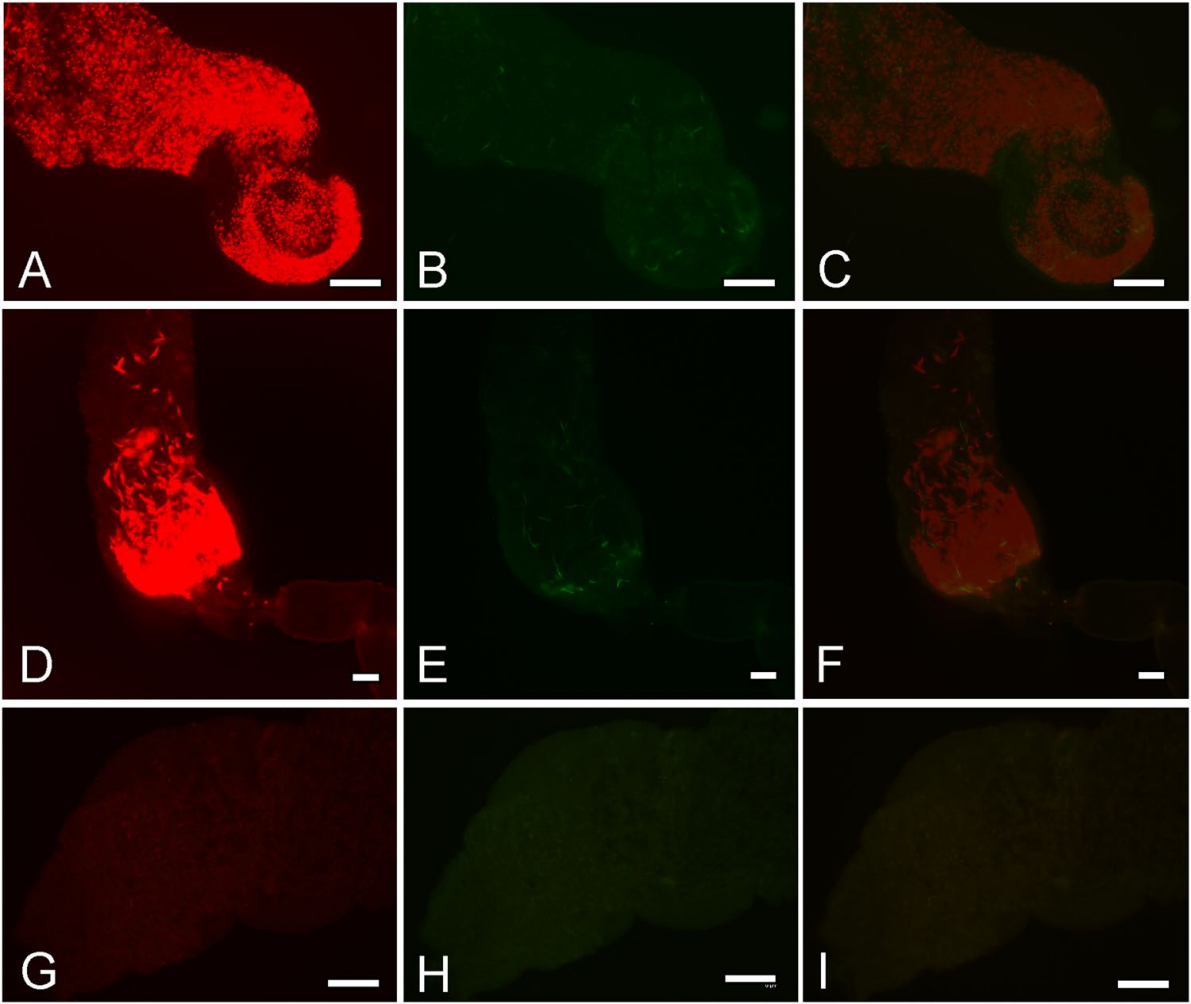
Fluorescence micrographs of thoracic midguts with cardia section and stomodeal valve of *Lutzomyia* spp. females on days 8 postinfection. Cardia of *Lu. longipalpis* (**A**–**C**) and *Lu. migonei* (**D**–**F**) coinfected by *Leishmania* (*Viannia*) *braziliensis* (green) and *Leishmania* (*Leishmania*) *infantum* (red). Control uninfected gut of *Lu. longipalpis* (**G**–**I**). (**A,D,G**) images from red fluorescence, (**B,E,H**) images from green fluorescence and (**C,F,I**) merged images, scale bar: 5 mm.

Co-infection did not alter the dynamics of infection of the individual parasite species and did not favour or harm any of the parasite species. In co-infections *L*. (*L*.) *infantum* produced high infection rates (Fig. 1a) and colonized the stomodeal valve in high numbers (Fig. 2D–F). In contrast, *L*. (*V*.) *braziliensis* infections were limited to the midgut and hindgut and some co-infected females became negative for *L*. (*V*.) *braziliensis* (Fig. 1b).

### Experimental infections of *Lu. longipalpis*

In total, 457 *Lu. longipalpis* females were dissected. On day 2 PI, infection rates were high in all parasite-vector combination (83%) (Fig. 1c), with parasites present in the endoperitrophic space (Fig. 1d).

On day 5 PI, *L*. (*L*.) *infantum* developed more successfully than *L*. (*V*.) *braziliensis* (infection rates of 75% and 53%, respectively (*X*^2^ = 4.16, *df* = 1, *P* = 0.04; *X*^2^ = 15.9, *df* = 1, *P* < 0.01).

On day 8, most of the females of *Lu. longipalpis* infected with *L*. (*L*.) *infantum* had a heavy infection rate (75%), whereas in *L*. (*V*.) *braziliensis* parasites were less numerous and the infection rate (66%) was significantly lower (*X*^2^ = 38.09, *df* = 2, *P* < 0.01) (Fig. 2A–C). *L*. (*L*.) *infantum* parasites frequently colonized the stomodeal valve while *L*. (*V*.) *braziliensis* infections were mostly limited to in the midgut and pylorus region of the hindgut (Fig. 1d). The same differences in parasite localization were observed in coinfections.

Co-infection did not alter the dynamics of infection of the individual parasite species and did not favour or harm any of the parasite species and the same differences in parasite localization were observed in coinfections.

### Morphometric analysis of promastigotes from midgut smears

**Figure 3:**
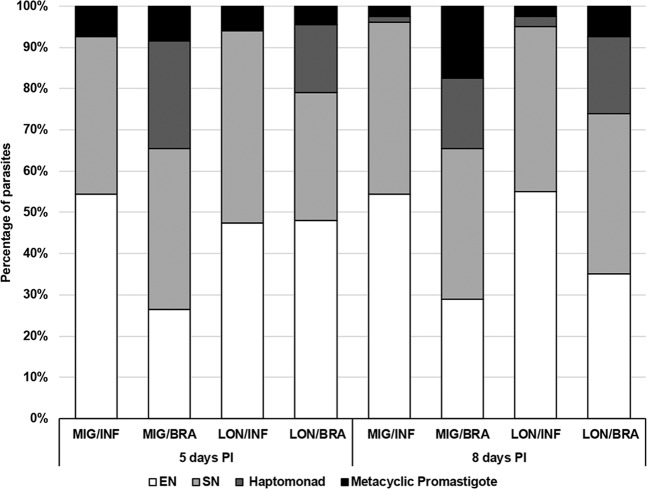
Morphological forms of *Leishmania* spp. during development in *Lutzomyia* spp. Morphological forms of *Leishmania* parasites in *Lutzomyia migonei* and *Lutzomyia longipalpis* were evaluated by light microscopy using oil-immersion objective on days 5 and 8 postinfection. MIG (*Lu. migonei*), BRA (*L*. (*V*.) *braziliensis*), LONG (*Lu. longipalpis*), INF (*L*. (*L*.) *infantum*), EN (elongated nectomonads), SN (short nectomonads).

**Figure 4:**
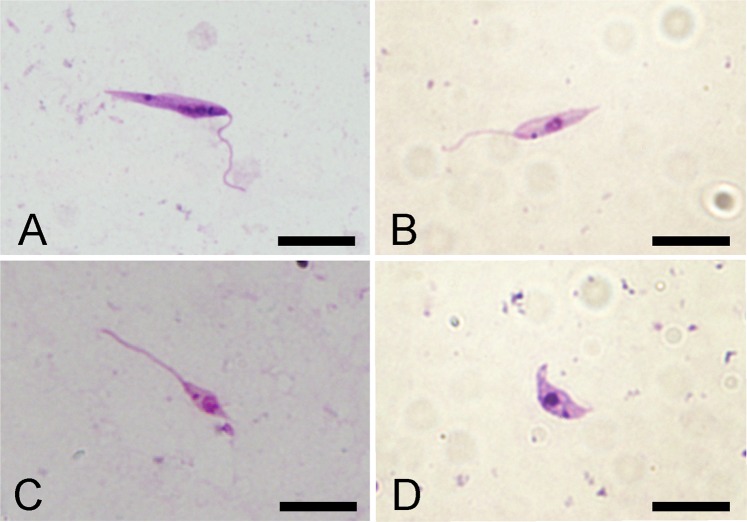
Morphological forms of *Leishmania* occurring in sand fly midgut of *Lu. migonei*. Scale bar 10 μm. (**A**) Elongated nectomonad of *L*. (*V*.) *braziliensis* in *Lu. migonei*; (**B**) Short nectomonad of *L*. (*L*.) *infantum* in *Lu. migonei* (**C**) Metacyclic promastigote of *L*. (*L*.) *infantum* in *Lu. migonei*; (**D**) haptomonads of *L*. (*L*.) *infantum* in *Lu. migonei*.

On day 5 PI, some differences in proportions of morphological forms in various parasite-vector combinations were documented. In *L*. (*L*.) *infantum*, elongated nectomonads were the prevailing forms in both vectors (55% *Lu. migonei* and 48% in *Lu. longipalpis*) (Fig. 3), and differences between various vectors were not significant (*X*^2^ = 3.00, *df* = 2, *P* = 0.22). On the other hand, significant differences were found between morphological forms of *L*. (*V*.) *braziliensis* (*X*^2^ = 20.95, *df* = 3, *P* < 0.01) in both vectors: elongated nectomonads (Fig. 4A) prevailed in *Lu. longipalpis* (48%) but short nectomonads (Fig. 4B) were the most numerous forms in *Lu. migonei* (39%). The metacyclic (Fig. 4C) forms were found in all parasite-vector combinations but in relatively low numbers, from 7.5 to 8.5% (Table 1).

**Table 1:** Promastigotes from gut smears were measured under light microscopy with an oil-immersion objective. MIG (*Lutzomyia migonei*). BRA (*Leishmania* (*Viannia*) *braziliensis*). LONG (*Lutzomyia longipalpis*). INF (*Leishmania* (*Leishmania*) *infantum*). PI (postinfection). EN (elongated nectomonads). SN (short nectomonads). HAP (haptomonads). MET (metacyclic promastigotes).

Day PI	Parasite-vector	Morphological form	*n*	Body length		Body width		Flagellar length	
				Mean (µm)	Range (µm)	Mean (µm)	Range (µm)	Mean (µm)	Range (µm)
5	MIG/INF	EN	109	17.3	25.9-14	3	5.2-1.5	23.8	33.8-17.1
		SN	76	11.9	13.9-7.3	3.1	5.9-1.6	13.11	25.3-3.2
		HAP	0	0	0	0	0	0	0
		MET	15	9.4	13.5-6.7	3.7	5.8-2.0	23.8	33.8-17.1
	MIG/BRA	EN	53	15.1	21.1-13.0	2.9	4.5-1.8	16.6	30.4-2.8
		SN	78	10.2	12.9-5.7	3.1	5.7-1.2	13.8	23.0-6.0
		HAP	52	9.7	12.8-3.5	3.4	5.9-1.9	5.8	12.1-1.1
		MET	17	9.1	11.8-6.2	3.5	4.5-2.0	20.8	25.5-14.8
	LON/INF	EN	95	17.4	26.4-14.0	2.3	3.7-1.3	19.1	29.2-5.8
		SN	93	11.3	13.9-6.0	2.2	4.3-1.2	13.2	26.6-2.0
		HAP	0	0	0	0	0	0	0
		MET	12	8.7	11.1-6.4	2.5	3.6-1.6	22.4	28.2-18.0
	LON/BRA	EN	96	13.2	28.3-16.3	2.9	12.2-1.2	18.4	31.8-3.5
		SN	62	10.7	12.8-5.7	2.9	6.9-1.3	15.4	23.4-7.5
		HAP	33	9.8	12.7-4.9	3	5.9-1.6	7.11	11.9-2.1
		MET	9	8.4	12.2-2.8	3.1	5.6-1.5	21.3	27.9-12.4
8	MIG/INF	EN	109	16.6	22.5-14	2.8	4.9-1.3	15.2	24.7-2.9
		SN	83	11.8	13.9-6.8	2.7	4.3-1.5	12.3	26.4-3.4
		HAP	3	12.9	13.9-11.5	3	3.1-2.9	2.7	2.9-2.4
		MET	5	8.6	9.5-7.6	2.8	3.3-2.0	19.5	24.7-16.9
	MIG/BRA	EN	58	15	18.9-13.1	2.9	17.2-1.5	20.9	30.6-2.9
		SN	73	10.8	12.9-5.8	2.4	3.7-1.5	17	24.6-10.2
		HAP	34	9.4	12.5-6.0	2.6	3.8-1.7	5.9	11.9-1.5
		MET	35	9	12.7-4.9	2.6	2.3-1.5	22.7	30.9-14.5
	LON/INF	EN	110	16.7	24.0-14.0	2.7	5.0-1.3	15.2	26.9-2.3
		SN	80	11.8	13.9-6.1	2.6	5.7-1.6	1.9	2.3-1.2
		HAP	5	11.3	12.2-9.8	2.3	2.8-1.9	11.4	24.6-3.0
		MET	5	9.6	13.2-6.9	3.6	5.1-2.2	23.4	31.1-23.4
	LON/BRA	EN	70	14.6	21.2-9.4	2.3	5.0-1.6	16.3	30.6-3.6
		SN	78	10.6	12.9-5.5	2.3	5.0-1.3	15.2	23.7-6.2
		HAP	37	10.6	12.7-6.5	2.2	3.5-1.2	6.4	23.7-1.8
		MET	15	10.2	12.9-4.5	2	3.1-1.1	19.9	24.5-15.2

On day 8 PI, differences between *Leishmania* species were more pronounced: in *Lu. migonei* and *Lu. longipalpis* infected by *L*. (*L*.) *infantum* the majority of parasites were present as elongated nectomonads, whereas in *Lu. migonei* and *Lu. longipalpis* infected by *L*. (*V*.) *braziliensis* the prevailing forms were short nectomonads (Fig. 3). Metacyclic forms were found in all parasite-vector combinations, the highest proportion (17.5%) were found in *Lu. migonei* infected by *L*. (*V*.) *braziliensis* (Table 1).

### Comparison of growth curves *in vitro*

**Figure 5:**
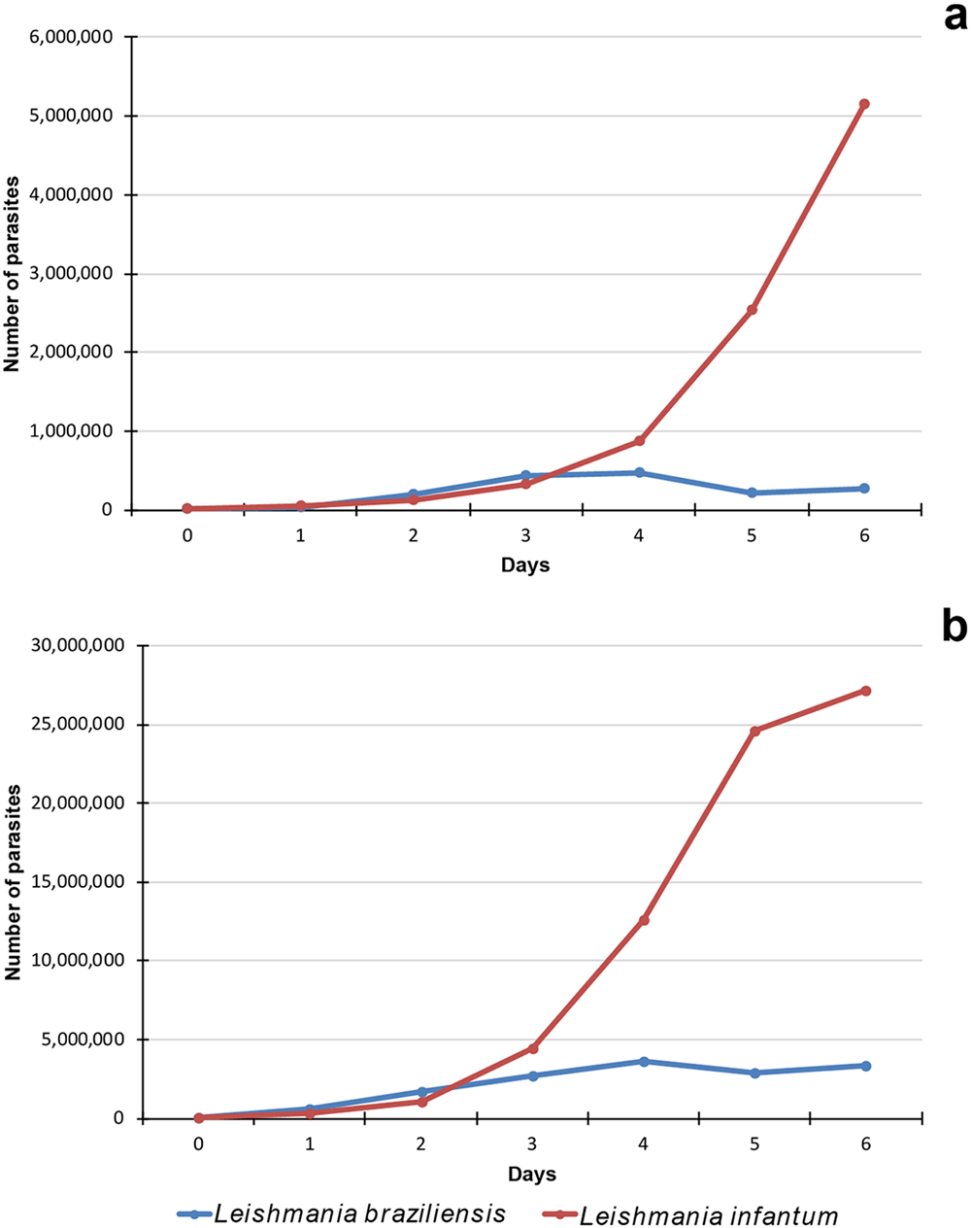
The curve growth of *Leishmania* spp. cultures at days 0, 1, 2, 3, 4, 5 and 6. Two initial concentrations were used for each *Leishmania* species. (**a**) Initial dose (at day 0) of 10^4^ promastigotes/ml; (**b**) Initial dose (at day 0) of 10^5^ promastigotes/ml.

To investigate the *in vitro* growth of *Leishmania* spp., the cultures were maintained at a temperature 26 °C. For both parasite species, the initial doses were 10^4^ promastigotes/ml and 10^5^ promastigotes/ml and the cells were cultivated for seven days. The growth of the parasites in culture was similar to that occurring in the phlebotomine sand flies: *L*. (*L*.) *infantum* and *L*. (*V*.) *braziliensis* grew similarly until the third day of cultivation (Fig. 5a,b), there were no statistical differences (the initial dose of 10^4^: *X*^2^ = 2.46, *df* = 3, *P* = 0.48, the initial dose of 10^5^: *X*^2^ = 6.77, *df* = 3, *P* = 0.08). From day 4 PI, *L*. (*V*.) *braziliensis* grew slower compared to *L*. (*L*.) *infantum*. The differences were statistically significant (the initial dose 10^4^: *X*^2^ = 256.2, *df* = 6, *P* < 0.01, the initial dose 10^5^: *X*^2^ = 95.1, *df* = 6, *P* < 0.01).

## Discussion

We evaluated the developmental patterns of two *Leishmania* spp. in two New World phlebotomine sand fly species, using both single infections and co-infections. Differences found between *L*. (*L*.) *infantum* and *L*. (*V*.) *braziliensis* were more pronounced than the differences between *Lutzomyia* species. In particular, *L*. (*L*.) *infantum* produced higher infection rates and grew more vigorously, as compared to *L*. (*V*.) *braziliensis*, in both *Lu. longipalpis* and *Lu. migonei*. Both sand fly species tested were similarly susceptible to infection by *L*. (*L*.) *infantum*, which confirms previous findings^11^.

Sand fly vectors have been classified into two categories, specific vectors and permissive vectors, based on their ability to support late-stage development of different *Leishmania* species^8,9^. Specific vectors, like *Phlebotomus papatasi* support development of a single parasite species or two closely related parasite species^6,12^ In contrast, permissive vectors support experimental infections by a broad range of *Leishmania* spp. The most important example of a permissive vector is *Lu. longipalpis*, which was involved in the establishment of *L*. (*L*.) *infantum* in Latin America^9^ and is proven vector of this parasite in many countries, from Costa Rica to northern Argentina^5,13^. Here we confirmed that *Lu. migonei* should be also considered as the permissive vector, as females of this species support the development of all *Leishmania* species tested so far, namely *Leishmania* (*Leishmania*) *amazonensis*, *L*. (*V*.) *braziliensis* and *L*. (*L*.) *infantum*^10,11^.

Nevertheless, our detailed comparative study revealed that *Lu. migonei* was more susceptible to *L*. (*V*.) *braziliensis* than *Lu. longipalpis*. Previously, several sand fly species were described as vectors of *L*. (*V*.) *braziliensis*^14^, but the role of *Lu. longipalpis* and *Lu. migonei* in the circulation of this parasite was unknown^5^. Natural infections of *Lu. migonei* by *L*. (*V*.) *braziliensis* were repeatedly found in the Rio de Janeiro State^10,15^. Nieves and Pimenta^10^ conducted a laboratory study on susceptibility of *Lu. migonei* to *L*. (*V*.) *braziliensis* resulting in 77% positivity of the dissected females. *Lu. longipalpis* carrying DNA of *L*. (*V*.) *braziliensis* has been described in the South-Eastern region of Brazil^16^ and in laboratory conditions 70% of *Lu. longipalpis* females developed late stage infections of *L*. (*V*.) *braziliensis*^17^.

In single infections and co-infections, *L*. (*L*.) *infantum* developed more rapidly in both sand fly species than *L. braziliensis*. This correlates with growth curves we observed *in vitro*: during initial three days both species grew similarly, but then, from day 3 onwards, *L*. (*L*.) *infantum* grew significantly faster. These results suggest that development in the sand fly is affected not only by the susceptibility of the vectors, but also by the growth characteristics of the studied strains. In late-stage infections, *L*. (*L*.) *infantum* was present in all midgut regions, the cardia and stomodeal valve in both sand fly species from day 5 PI, showing typical suprapylarian type of development. In contrast, *L*. (*V*.) *braziliensis* was concentrated in the hindgut and the abdominal midgut (peripylarian development). The same happens in the co infections, demonstrating that the two parasites conclude their development and do not compete with each other. Previous study with *Lu. migonei* and two *Leishmania* species was done using single infections only, but observations by Nieves and Pimenta^10^ were similar to our results: suprapylarian *L*. (*L*.) *amazonensis* grew faster than peripylarian *L*. (*V*.) *braziliensis*.This interspecific difference appears to be strain-independent as almost identical patterns of development were described for the *L. (V.) braziliensis* strain m2903^10^ and for two different *L*. (*L*.) *infantum* strains M4192 and CUK3 in *Lu. migonei*^11^.

From an epidemiological point of view, it is very important to carry out studies, assessing the development of two species of parasites that cause different forms of leishmaniasis, especially when they coexist in endemic regions^18^. Both *Leishmania* species completed the life cycle, producing infective forms in both sand fly species studied. We conclude that both *Lu. migonei* and *Lu. longipalpis* are equally susceptible vectors for *L*. (*L*.) *infantum*, in laboratory colonies. In relation to *L*. (*V*.) *braziliensis*, *Lu. migonei* appears to be more susceptible to this parasite than *Lu. longipalpis*.

## Material and Methods

### Sand fly colonies and *Leishmania* strains

Established laboratory colonies of *Lu. longipalpis* (from Jacobina, Brazil) and *Lu. migonei* (from Baturité, Brazil) were used and maintained under standard conditions, as previously described^19^. A fluorescent strains of *L*. (*V*.) *braziliensis* (XB29 marked with GFP) and *L*. (*L*.) *infantum* (OGVL marked with mCherry) by stable integration of mCherry into the 18 S rRNA locus as previously described^20^. We have used parasites within less than 10 passages *in vitro* and were maintained at 23 °C on Medium 199 (Sigma-Aldrich, USA) supplemented with 10% fetal calf serum (Thermo Fisher Scientific, USA), 1% BME vitamins (Sigma-Aldrich), 2% human urine and 250 μg/ml amikin (Bristol-Myers Squibb, USA).

For study of growth curves *in-vitro*, two initial doses were used: 10^4^ promastigotes/ml and 10^5^ promastigotes/ml. The concentration of parasites was analyzed daily for 7 days, using counting in Burker chamber. The experiments were repeated twice.

### Experimental infections of sand flies

Female sand flies (3–6 days old) of both species were fed through a chick-skin membrane on heat-inactivated rabbit blood containing 10^6^ promastigotes/ml for single infections and 5 × 10^5^ of each species for co-infections (half a dose used in single infections). Three groups of females of *Lu. migonei* were studied, the first infected with *L*. (*V*.) *braziliensis* (XB29 marked with GFP), the second with *L*. (*L*.) *infantum* (OGVL marked with mCherry) and the third co-infected with both *Leishmania* species. The same procedure was performed with *L. longipalpis*. Engorged females were separated and maintained in the same conditions as the colony and dissected on days 2, 5 and 8 post-infection (PI). Individual guts were placed into a drop of saline and analyzed by fluorescence microscopy for the localization, infection intensity and morphology of *Leishmania* infections. Parasite loads were graded according to Myskova *et al*.^21^ as light (<100 parasites per gut), moderate (100 to 1000 parasites per gut) and heavy (>1000 parasites per gut). The experiments were repeated four times.

### Morphometry of parasites

Smears from midguts of *Lu. migonei* and *Lu. longipalpis* infected with *L*. (*L*.) *infantum* and *L*. (*V*.) *braziliensis*, alone or in combination (i.e., co-infected) were prepared on days 5 and 8 PI, being fixed with methanol and stained with Giemsa. Stained smears were then examined under a light microscope with an oil-immersion objective and photographed with an Olympus D70 camera (Olympus, Hong-Kong, China). Body length, body width and flagellar length of 200 randomly selected promastigotes from four females/smears were measured for each sand fly species and time intervals using Image-J 1.x software. Promastigote forms were distinguished according to Walters *et al*.^22^ and Sadlova *et al*.^23^. *L*. (*L*.) *infantum*, developmental forms were identified as follows: (i) elongated nectomonads (body length ≥14 μm) (ii) short nectomonads (body length <14 μm and flagellar length ≤2 times body length); (iii) metacyclic promastigotes (body length <14 μm and flagellar length ≥2 times body length); and (iv) haptomonads (flagellum 0–3 µm, present during the late stage infections in cardia). *L*. (*V*.) *braziliensis*, developmental forms were identified as follows: (i) elongated nectomonads (body length ≥13 μm); (ii) short nectomonads (body length <13 μm and flagellar length  ≤ 2 times body length); (iii) metacyclic promastigotes (body length <13 μm and flagellar length ≥2 times body length); and (iv) haptomonads (flagellar length ≤ body length, present in hind gut and cardia).

### Ethical approval

Animals used for blood-feeding of sand fly colonies were maintained and handled in the animal facility of Charles University in Prague, in accordance with institutional guidelines and Czech legislation (Act of the Czech National Assembly on the Protection of Animals Against Cruelty No. 246/1992, latest amendment No. 359/2012), which complies with all relevant European Union guidelines for experimental animals. All experiments were approved by the Committee on the Ethics of Laboratory Experiments of the Charles University in Prague and were performed under the Certificate of Competency (Registration Number: CZ 03069).
